# Inward- and outward-facing homology modeling of human concentrative nucleoside transporter 3 (hCNT3) predicts an elevator-type transport mechanism

**DOI:** 10.1080/19336950.2018.1506665

**Published:** 2018-08-28

**Authors:** Sylvia Y. M. Yao, James D. Young

**Affiliations:** Membrane Protein Disease Research Group, Department of Physiology, University of Alberta, Edmonton, Canada

**Keywords:** Elevator-type transport mechanism, human concentrative nucleoside transporter 3 (hCNT3), nucleoside transport, substituted cysteine accessibility (SCAM) analysis

## Abstract

The human SLC28 family of concentrative (Na^+^-dependent) nucleoside transporters has three members, hCNT1, hCNT2 and hCNT3. Previously, we have used heterologous expression in *Xenopus laevis* oocytes in combination with an engineered cysteine-less hCNT3 protein hCNT3(C-) to undertake systematic substituted cysteine accessibility method (SCAM) analysis of the transporter using the membrane-impermeant thiol reactive reagent *p*-chloromercuribenzene sulfonate (PCMBS). A continuous sequence of more than 300 individual amino acid residue positions were investigated, including the entire transport domain of the protein, as well as important elements of the corresponding hCNT3 structural domain. We have now constructed 3D structural homology models of hCNT3 based upon inward-facing, intermediates and outward-facing crystal structures of the bacterial CNT *Neisseria wadsworthii* CNT_NW_ to show that all previously identified PCMBS-sensitive residues in hCNT3 are located above (*ie* on the extracellular side of) the key diagonal barrier scaffold domain TM9 in the transporter’s outward-facing conformation. In addition, both the Na^+^ and permeant binding sites of the mobile transport domain of hCNT3 are elevated from below the scaffold domain TM9 in the inward-facing conformation to above TM9 in the outward-facing conformation. The hCNT3 homology models generated in the present study validate our previously published PCMBS SCAM data, and confirm an elevator-type mechanism of membrane transport.

Specialized nucleoside transporter (NT) proteins are required for passage of nucleosides and hydrophilic nucleoside analogs across biological membranes [1]. Physiologically, nucleosides serve as nucleotide precursors in salvage pathways [2]. Adenosine modulates diverse physiological processes through interaction with purinoreceptors [2]. Pharmacologically, nucleoside analogs are used as chemotherapeutic agents in the treatment of cancer and viral diseases [3]. Human (h) NT proteins belong to two families: the SLC28 concentrative nucleoside transporter (CNT) family and the SLC29 equilibrative nucleoside transporter (ENT) family [1]. hENTs are equilibrative nucleoside transporters that mediate bidirectional fluxes of purine and pyrimidine nucleosides down their concentration gradients, and are ubiquitously found in most, possibly all, cell types [1]. hCNTs are inwardly directed Na^+^-dependent nucleoside transporters found predominantly in intestinal and renal epithelial and other specialized cell types [1]. hCNT1 and hCNT2 are pyrimidine and purine nucleoside selective, respectively, and couple Na^+^:nucleoside cotransport with a 1:1 stoichiometry [4]. hCNT3 transports both pyrimidine and purine nucleosides, and couples Na^+^:nucleoside cotransport with 2:1 stoichiometry [5]. Unlike hCNT1 and hCNT2, hCNT3 is also capable of H^+^:nucleoside cotransport with a coupling stoichiometry of 1:1, whereby one of the two Na^+^ binding sites also functionally interacts with H^+^.

In 2012, the first crystal structure of a CNT, representing the inward-facing conformation of a bacterial Na^+^-linked CNT from *Vibrio cholera* (vcCNT), was determined at a resolution of 2.4 Å, the transporter having both one uridine molecule and one Na^+^ ion bound [6]. *Vibrio* CNT is homo-trimeric in membrane architecture and shows 39% amino acid sequence identity with hCNT3. Each protomer contains eight transmembrane-spanning α-helices (TM1-TM8), three interfacial helices (IH1-IH3) oriented parallel to the plane of the membrane, and two re-entrant helical hairpins (HP1 and HP2) that have opposite orientations in the membrane. The predicted membrane topology of homologous hCNT3 has extended extracellular C-terminal regions containing multiple sites of N-linked glycosylation, with an additional three TMs at its N-terminus that are not essential for transport activity [7]. By analogy with the bacterial structure, hCNT3 topology can be subdivided into an outer or “scaffold” domain comprising TM4, TM5, IH1, TM6 and TM9, which surrounds an inner or “transport” domain that can itself be divided into two structural subdomains that are related by an internal two-fold pseudo-symmetry (Figure 1(a)). The first transport subdomain comprises IH2, HP1, TM7 and TM8, while the second transport subdomain comprises IH3, HP2, TM10 and TM11. The nucleoside and Na^+^ binding sites in vcCNT are located side-by-side at the center of the two-fold pseudo-symmetry and are formed by the tips of HP1 and HP2, and the unwound regions of TM4 and TM7 (equivalent to TM7 and TM10 of hCNT3) [6].

Additional valuable information has been gained about residue localization and function in hCNT3 by substituted cysteine accessibility method (SCAM) analysis using a cysteine-less version of hCNT3 (hCNT3C-) and thiol-specific reagents such as *p*-chloromercuribenzene sulfonate (PCMBS). Measured as transport inhibition, reactivity of introduced cysteine residues in hCNT3C- to PCMBS, which is both membrane-impermeable and hydrophilic, indicates pore-lining status with access from the extracellular medium. The ability of permeant, such as uridine, to protect against this inhibition denotes location within, or closely adjacent to the outward-facing permeant-binding pocket. In a series of recently published [8] and previous studies [9,10], we have presented a comprehensive PCMBS SCAM analysis of 305 residues in hCNT3 spanning the region between and including IH2 and TM11. Incorporating the entire transport domain of hCNT3, as well as the linker region (TM9) between transport subdomains IH2-TM8 and IH3-TM11, our work identified 53 PCMBS-inhibitable residues, of which 25 were uridine protected. Notably, four of the uridine-protected amino acid residue positions (Gln341, Thr342, Asn565 and Ile571) were located deep within the transporter, and close to the cytoplasmic side of the membrane in a predicted inward-facing homology model of hCNT3 derived from the vcCNT crystal structure [8]. Three of these residues (Gln341, Thr342, and Asn565) are amongst the nine homologous residues in vcCNT implicated in uridine binding, while Ile571 lies closely adjacent in linear sequence to Asn565. We have also used a computer-generated repeat-swap outward-facing homology model of vcCNT to generate a corresponding outward-facing homology model of hCNT3 [8]. Relative to the modeled hCNT3 inward-facing conformation, the repeat-swap outward-facing conformation showed bound uridine to be elevated in the membrane, and more accessible to the extracellular milieu [8].

Alternating-access mechanisms of membrane transport have been grouped into three categories: the well-established rocker switch- and rocking bundle-type mechanisms, and the newer and novel elevator-type mechanism [11]. Most transporters employ the rocker switch or rocking bundle types of transport mechanism, in which permeant binds between two structurally similar or dissimilar domains of the transport protein, catalyzing a conformational change around the centrally located permeant-binding site to alternately expose the binding pocket to intracellular and extracellular sides of the membrane [11]. The third and most recently described category of alternating-access membrane transport is the elevator-type mechanism. Permeant first binds to the mobile transport domain of the protein, which then undergoes a large rigid-body movement against the relatively immobile scaffold domain of the transporter to translocate the bound permeant to the other side of the membrane. Therefore, unlike the rocker switch or rocking bundle mechanisms, in which two domains move around a centrally-located permeant binding site for alternating membrane side accessibility, proteins with an elevator-type transport mechanism use the transport domain to move the permeant across the membrane [11]. Elevator-type transport mechanisms have been demonstrated crystallographically for the *Pyrococcus horikoshii* Na^+^-aspartate symporter Glt_ph_, the *Yersinia frederiksenii* Na^+^-dependent bile-acid symporter SBT, the *Thermus thermophilus* Na^+^/H^+^ antiporters NapA, and the *Klebsiella pneumoniae* Na^+^/citrate symporter CitS [12–15]. In each of these cases, both inward-facing and outward-facing crystal structures were required to reveal that the transporter function by an elevator-type mechanism. The bacterial homologue of hCNT3, vcCNT, was crystalized in the inward-facing conformation [6], with an inverted repeat architecture resembling Glt_ph_. Both vcCNT and Glt_ph_ are homotrimers that consist of a scaffold domain and a transport domain, the latter in both cases containing the key structural elements of the permeant binding site made up of helical hairpins and discontinuous helices [6,12].

At the same time as we published our most recent hCNT3 SCAM paper [8], Lee and co-workers determined multiple crystal structures with different conformational states along the transport cycle of a second bacterial CNT from *Neisseria wadsworthii* (CNT_NW_) [16]. Like vcCNT, CNT_NW_ is 38% identical in amino acid sequence to hCNT3. The CNT_NW_ crystal structures included inward-facing open and inward-facing occluded states, various intermediate states, and the outward-facing open state. The inward-facing occluded (uridine and Na^+^ bound) crystal structure of CNT_NW_ is nearly identical to the vcCNT crystal structure, with slightly reassigned scaffold and transport domains compared to the original arrangement for vcCNT [6], (Figure 1(b)). The scaffold domain of CNT_NW_ comprises TM3 and TM6, plus IH1, IH2 and IH3 (equivalent to TM6, TM9, IH1, IH2 and IH3 of hCNT3) in conjunction with a transport domain composed of TM1, TM2, TM4, TM5, TM7, TM8, HP1, and HP2 (equivalent to TM4, TM5, TM7, TM8, TM10, TM11, HP1 and HP2 of hCNT3), the latter containing both the Na^+^ and permeant (nucleoside) binding sites. The newly revealed CNT_NW_ crystal structures provide confirmatory evidence that bacterial CNTs function through an elevator-type mechanism, and define the conformational transitions involved [16]. In comparison with the inward-facing conformation, the outward-facing conformation of CNT_NW_ reveals a structure in which the scaffold domain is largely unchanged, while the transport domain moves ~12 Å towards the extracellular side of the membrane, placing the nucleoside (and Na^+^) binding sites above TM6 (equivalent to TM9 of hCNT3), and even more accessible to the extracellular medium than in the predicted outward-facing repeat-swap homology model of hCNT3 [8,16].

**Figure 1.**
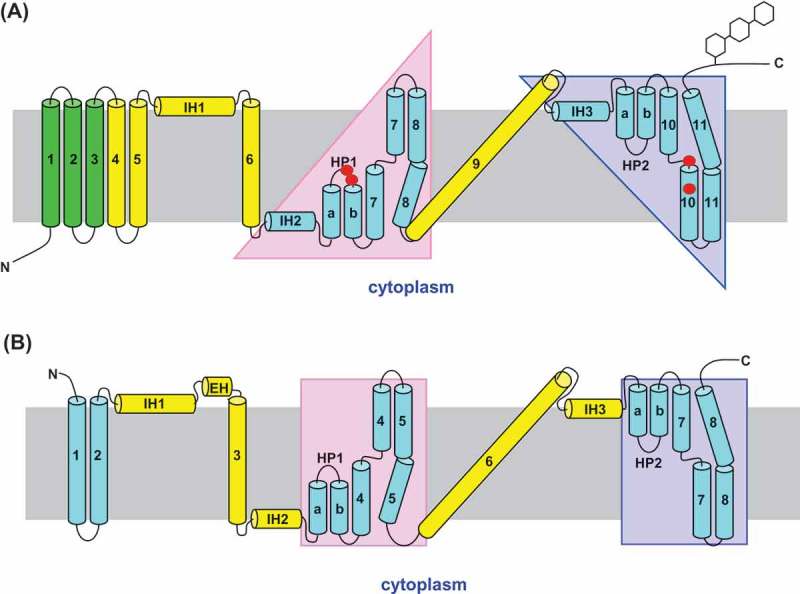
Topological models of hCNT3 and CNT_NW_. (a) Predicted membrane topology of human concentrative nucleoside transporter hCNT3 based upon that of its bacterial counterpart vcCNT from *Vibrio cholera*. PCMBS sensitive and uridine-protected residues Gln341, Thr342, Asn565 and Ile571 are shown as *red circles*. (b) Membrane topology of *Neisseria wadsworthii* CNT (CNT_NW_) [16]. The hCNT3 N-terminal transmembrane helices TM1, TM2 and TM3 and C-terminal extramembranous tail with glycosylation sites are not present in the bacterial protein.

Lee and co-workers [16] also identified additional 3D forms of CNT_NW_ in the absence of sodium and uridine whose structures were intermediate between the inward-facing and outward-facing conformations, referring to them as CNT_NW_ intermediate 1, intermediate 2 and intermediate 3, with intermediate 1 closest in structure to the inward-facing conformation, and intermediate 3 closest to the outward-facing conformation. Placing each of the five CNT_NW_ structures in sequence, it was shown that critical transport domain component HP1 moved ~9Å from the inward-facing conformation through the three intermediate structures, and then ~5Å from intermediated 3 to the outward-facing conformation, a total transmembrane distance of ~14 Å [16].

For the purposes of the present addendum to our most recent SCAM paper [8], and to more precisely model the outward-facing conformation of hCNT3 and its proposed elevator-type mechanism of transport, we have constructed 3D structural homology models of hCNT3 based upon each of the five following CNT_NW_ crystal structures: CNT_NW_ (inward-facing, open), CNT_NW_ intermediate 1, CNT_NW_ intermediate 2, CNT_NW_ intermediate 3, and CNT_NW_ (outward-facing, open). Models were produced using the program SWISS-MODEL [17] and Protein Data Bank entries 5L2A protomer A, 5L27 protomer C, 5L24 protomer C, 5U9W protomer C, and 5L2A protomer C, respectively.

Figure 2(a,b) show the resulting homology model of hCNT3 in its outward-facing open conformation. Figure 2(a) highlights in *red* the side-chains of PCMBS-sensitive residues; Figure 2(b) highlights in *red* the side chains of residues that are both PCMBS-sensitive and uridine-protected. In our previously published SCAM paper [8], we showed that all 53 hCNT3 PCMBS-sensitive residues (of which 25 are uridine-protected) cluster around and below the scaffold domain TM9 in the inward-facing homology model of hCNT3. In marked contrast, however, in the present outward-facing homology model of hCNT3, all of the PCMBS-sensitive residues are located above TM 9 (Figure 2(a,b)), consistent with an upward movement of the transport domain and associated nucleoside binding site to become more accessible to the extracellular side of the membrane. Figure 3 shows in reverse order of cellular nucleoside uptake all five modeled states of hCNT3, identifying the specific changing locations of the PCMBS-sensitive, uridine protected residues Gln341, Thr342, Asn565 and Ile571. Gln341, Thr342 and Asn565 are implicated in uridine binding, with the amino acid side-chains of Gln341 and Thr342 located at the tip of HP1 and interacting with the uracil base, and Asn565 located in the unwound region of TM10 and interacting with the ribose moiety of uridine [6]. Ile571 is likely not directly involved in uridine binding, but is located in TM 10 close to Asn565. In the inward-facing conformational state, scaffold domain TM9 closes off the binding pocket containing Gln341, Thr342, Asn565 and Ile571 from the extracellular side of the membrane. In the intermediate states, the nucleoside binding site moves upwards to the center of the membrane, and is occluded from both sides of the membrane. In the outward-facing state, residues Gln341, Thr342, Asn565 and Ile571 are now above TM 9, exposed to the extracellular side of the membrane and accessible for reaction with PCMBS.

**Figure 2.**
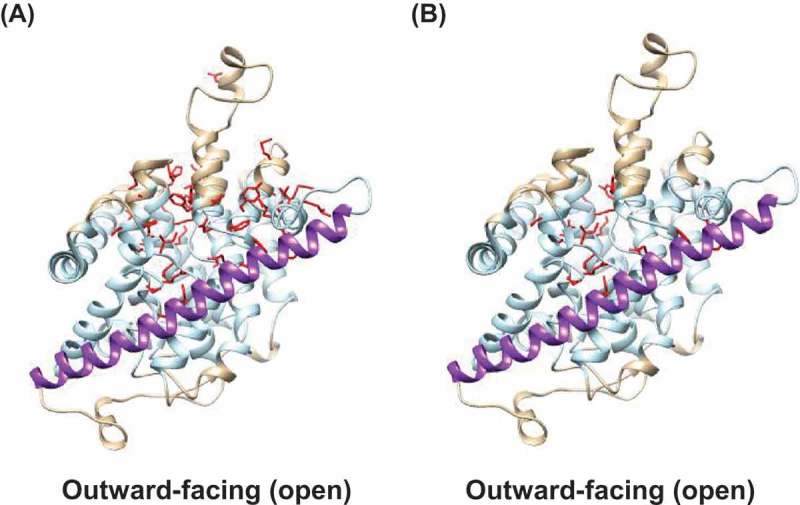
Homology models of hCNT3. Cartoon representations of the hCNT3 3D outward-facing conformation based upon the corresponding crystal structure of the bacterial nucleoside transporter CNT_NW_ (Protein Data Bank entries 5L2A, protomer C) using the program SWISS-MODEL [17] and viewed parallel to the membrane. Molecular graphics and analyzes were performed using the UCSF Chimera package [18]. The extracellular boundaries of the hydrophobic core of the lipid bilayer were predicted using the PPM server [19], with residues outside of the hydrophobic boundaries of the bilayer shown in *beige*, and residues within the hydrophobic core of the bilayer shown in *light blue*. TM9 of the outer scaffold domain of hCNT3 is shown in *purple*. (A) Outward-facing homology model of hCNT3 with side-chains of PCMBS-sensitive residues shown in *red*. (B) Outward-facing homology model of hCNT3 with side-chains of PCMBS-sensitive and uridine-protected residues shown in *red.*

Extending the analysis to cation binding, hCNT3 has two Na^+^ binding sites, with only one of these (the primary Na^+^ binding site) identified in the crystal structures of vcCNT and CNT_NW_. Figure 4(a) shows this primary Na^+^ binding site in the predicted inward-facing and outward-facing conformations of hCNT3 based upon the crystal structures of CNT_NW_. Side chains of contributing amino acid residues, Asn336, Val339, Thr370, and Ile371 are highlighted in *blue*. Figure 4(b) shows this Na^+^ binding site (again highlighted in *blue*) in the two conformations together with the side chains of adjacent residues (Gln341, Thr342, Glu343, Glu519, Asn565, Ser568 and Phe563) contributing to the permeant (nucleoside) binding site (highlighted in *red*). Both the Na^+^ and permeant binding sites of the hCNT3 transport domain are elevated from below the scaffold domain TM9 in the inward-facing conformation to above TM9 in the outward-facing conformation.

**Figure 3.**
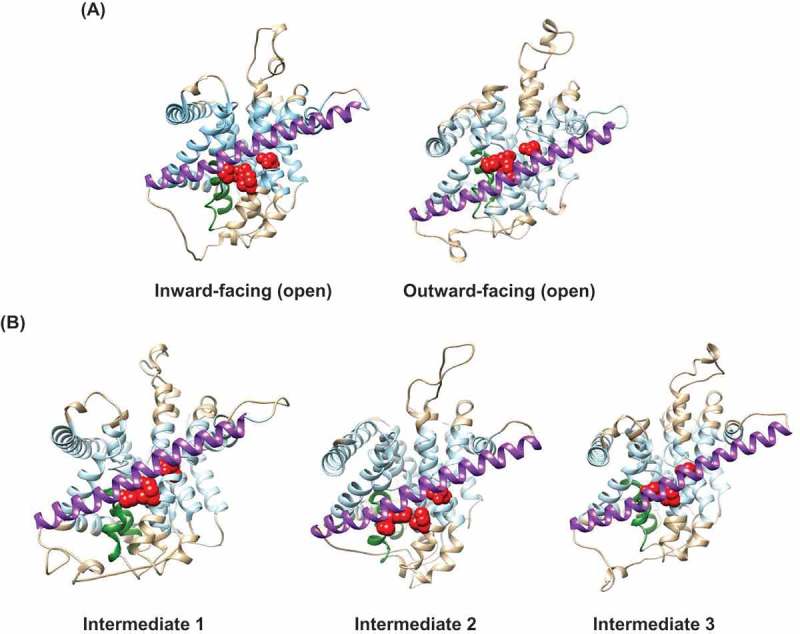
Homology models of hCNT3 in different conformational states of the transport cycle. Cartoon representations of hCNT3 3D models are based upon the corresponding crystal structures of the bacterial nucleoside transporter CNT_NW_ using the program SWISS-MODEL [17] and viewed parallel to the membrane. The extracellular boundaries of the hydrophobic core of the lipid bilayer were predicted using the PPM server [19], with residues outside of the hydrophobic boundaries of the bilayer shown in *beige*, and residues within the hydrophobic core of the bilayer shown in *light blue*. (a) Inward-facing, open state (Protein Data Bank entry 5L2A, protomer A) and outward-facing, open state (Protein Data Bank entry 5L2A, protomer C). (b) Intermediate-1 (Protein Data Bank entry 5L27, protomer C); Intermediate-2 (Protein Data Bank entry 5L24, protomer C); Intermediate-3 (Protein Data Bank entry 5U9W, protomer C). Side chains of PCMBS-sensitive, uridine-protected residues Gln341, Thr342, Asn565 and Ile571 are shown as *red spheres*. HP1 (residues 327 to 350 of hCNT3) are shown in *green*. TM9 of the outer scaffold domain (residues 432 to 475 of hCNT3) is shown in *purple.*

**Figure 4.**
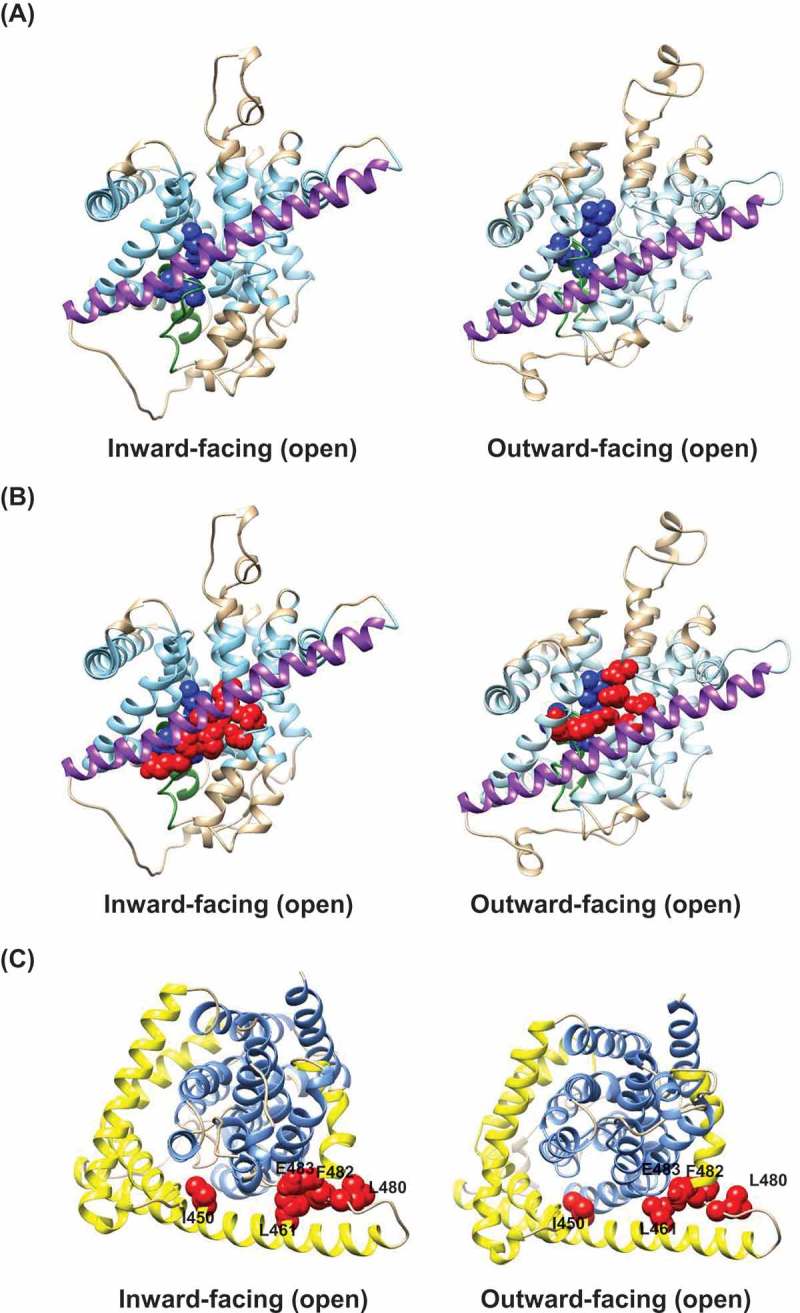
Inward-facing and outward-facing homology models of hCNT3. Cartoon representations of hCNT3 3D inward-facing and outward-facing conformations are based upon the corresponding crystal structures of bacterial nucleoside transporter CNT_NW_ (Protein Data Bank entries 5L2A, protomer A and protomer C, respectively) (a) Models of hCNT3 viewed parallel to the membrane. Side chains of residues involved in primary Na^+^-binding site are shown as *blue spheres*. (b) Models of hCNT3 viewed parallel to the membrane. Side chains of residues involved in primary Na^+^-binding site and substrate-binding site are shown as *blue* and *red spheres*, respectively. The extracellular boundaries of the hydrophobic core of the lipid bilayer were predicted using the PPM server [19], with residues outside of the hydrophobic boundaries of the bilayer shown in *beige*, and residues within the hydrophobic core of the bilayer shown in *light blue*. TM9 of the outer scaffold domain is shown in *purple*. (c) Models of hCNT3 viewed from the extracellular surface of the membrane. The outer scaffold domain of hCNT3 (TM6, TM9, IH1, IH2 and IH3) and inner transport domain of hCNT3 (TM4, TM5, HP1, TM7, TM8, HP2, TM10 and TM11) are shown in *yellow* and *blue*, respectively. Side chains of residues (Ile450 and Leu461) and (Leu480, Phe482 and Glu483) from the scaffold domain TM9 and linker region to IH3, respectively, are highlighted as *red spheres.*

The primary Na^+^ binding site of hCNT3 also functionally interacts with H^+^, possibly in the form of a hydronium ion. Residues Gln341, Thr342, Asn565 and Ile571 of hCNT3 are PCMBS-sensitive and uridine-protected in both Na^+^-containing, H^+^-reduced medium and in Na^+^-free, acidified medium [8,9]. However, we have also identified some hCNT3 residues which are PCMBS-sensitive only under Na^+^-containing, H^+^-reduced conditions, and some that are PCMBS-sensitive only under Na^+^-free, acidified conditions [8,9]. This implies that at least two subtly different outward-facing conformations of hCNT3 may exist, depending on the cation milieu. The nature of these differences remains to be identified, but have important functional consequences: the Na^+^-coupled transporter is broadly selective for both purine and pyrimidine nucleosides and nucleoside drugs, whereas the proton-coupled transporter is uridine specific [5]. An additional question is the location of the second hCNT3 Na^+^ binding site. Although not based upon crystallographic data, site-directed mutagenesis studies have potentially identified residues implicated in the second Na^+^ binding site [20]. For example, our SCAM analyzes found that conversion of Thr605 in TM11 of transport domain to cysteine changed the flux ratio of Na^+^-mediated *versus* H^+^-mediated uridine uptake from 1.7 for wild-type hCNT3 to 0.6 [8]. Further studies of this and other such residues may assist location of the second Na^+^ binding site of hCNT3.

Finally, we considered two adjacent regions of the hCNT3 scaffold domain that are also critically important to transport function, TM9 and the flexible linker region between it and IH3. In our PCMBS SCAM analysis of hCNT3 [8], we identified two PCMBS-sensitive residues in TM9 (Ile450 and Leu461) and three in the linker region between TM9 and IH3 (Leu480, Phe482 and Glu483). In particular, Leu480, Phe482 and Glu483 are concentrated in a short region of 8 amino acids forming the possible flexible hinge region in the scaffold domain. Figure 4(c) shows the modeled locations of the five PCMBS-sensitive residues in both inward-facing and outward-facing conformations of hCNT3. PCMBS-induced transport inhibition at these residue positions, none of which were protected by uridine, is consistent with PCMBS involvement in restricting the upward or downward movements of the transport domain and interrupting the transport cycle, either by obstructing movement of the transport domain (Ile450 and Leu461) or by direct interference with hinge function (Leu480, Phe482 and Glu483).

In summary, therefore, the hCNT3 homology models generated in the present study validate our previously published PCMBS SCAM data and *vice versa* and, additionally, confirm transport by an elevator-type mechanism.^1^

## Note

1. The abbreviations used are: NT, nucleoside transporter; CNT, concentrative nucleoside transporter; ENT, equilibrative nucleoside transporter; TM, putative transmembrane domain; SCAM, substituted cysteine accessibility method; IH, interfacial helix; HP, helical hairpin; PCMBS, *p*-chloromercuribenzene sulfonate.
